# AI ethics in computational psychiatry: From the neuroscience of consciousness to the ethics of consciousness

**DOI:** 10.1016/j.bbr.2021.113704

**Published:** 2022-02-26

**Authors:** Wanja Wiese, Karl J. Friston

**Affiliations:** a Institute of Philosophy II, Ruhr University Bochum, Universitätsstraße 150, 44780 Bochum, Germany; b Wellcome Centre for Human Neuroimaging, University College London, 12 Queen Square, London WC1N 3AR, UK

**Keywords:** AI ethics, Computational psychiatry, Consciousness, Ethics of consciousness, Mental disorders, Schizophrenia

## Abstract

Methods used in artificial intelligence (AI) overlap with methods used in computational psychiatry (CP). Hence, considerations from AI ethics are also relevant to ethical discussions of CP. Ethical issues include, among others, fairness and data ownership and protection. Apart from this, morally relevant issues also include potential *transformative effects* of applications of AI—for instance, with respect to how we conceive of autonomy and privacy. Similarly, successful applications of CP may have transformative effects on how we categorise and classify mental disorders and mental health. Since many mental disorders go along with disturbed conscious experiences, it is desirable that successful applications of CP improve our understanding of disorders involving disruptions in conscious experience. Here, we discuss prospects and pitfalls of transformative effects that CP may have on our understanding of mental disorders. In particular, we examine the concern that even successful applications of CP may fail to take all aspects of disordered conscious experiences into account.

## Highlights

• Considerations from AI ethics are also relevant to the ethics of computational psychiatry.
• Ethical issues include, among others, fairness and data ownership and protection.
• They also include potential *transformative effects*.
• Computational psychiatry may transform conceptions of mental disorders and health.
• Disordered conscious experiences may pose a particular challenge.

## 1 Introduction

Methods used in *computational psychiatry* (CP) [5], [6], [7], [1], [2], [3], [4], such as deep learning, Bayesian modelling, or reinforcement learning, overlap with methods used in artificial intelligence [8]. Although the methods may be used for different aims, they can raise similar ethical issues. Hence, considerations from AI ethics are also relevant to CP. For instance, algorithms may produce unfair outcomes if their training data are biased [9] and the possibility to collect and analyse personal data using algorithms raises issues of data ownership and protection [11], [10]. Furthermore, many applications of AI are not explicable, i.e., it is often difficult or impossible to determine why an AI system yields a given outcome or who is accountable for the particular way in which an AI system works [12]. Such immediate ethical concerns arise for applications of AI in general, but also for applications in mental healthcare and CP in particular [15], [13], [14].

In addition to such immediate concerns, applications of AI can have morally relevant *transformative effects*. We use the term “transformative effects” broadly, in the sense of persistent changes that significantly impact human well-being related to at least some aspects of life and society. These changes need not be extreme or radical (in the sense of *transformative AI*, [16]), nor need they fundamentally change personal preferences (in the sense of *transformative experience*, [17]). Transformative effects can still be far-reaching and substantial, for instance, by affecting the way we conceive of autonomy and privacy, or by transforming our way of living through AI applications that permeate daily life [18]. Similarly, successful applications of CP may transform how we classify and define mental disorders [7], [1], [3], [19], which can have direct and indirect consequences for the well-being of affected persons [20]. Many mental disorders are characterised by disturbed conscious experience. We shall refer to such disorders as “disorders of consciousness.”

The term “disorder of consciousness” is often reserved for disordered global states of consciousness, such as unresponsive wakefulness syndrome, minimally conscious state, or coma [21], [22]. In these conditions, wakefulness and awareness are either diminished (minimally conscious state), partially absent (unresponsive wakefulness) or jointly absent (coma). Here, we use the term in a broader sense, which also covers disorders involving a disruption of the contents or the structure or form of conscious processes (including their spatiotemporal continuity, see [23]). Examples include hallucinations in psychosis [24], deviant time- and self-consciousness in major depressive disorder [25], depersonalisation [26], and derealisation in schizophrenia [27]. These conditions need not go along with diminished levels of wakefulness or awareness; still, they are characterised by disruptions of conscious processing. For this reason, it will be useful to refer to them as *disorders of consciousness* in this paper.

By “consciousness” we mean *phenomenal consciousness*, i.e., mental states for which there is something it is like to be in [28]. These qualitative aspects can include consciously experienced positive or negative affect, suffering, bodily feelings, cognitive phenomenology, as well as temporal, spatial, and other perceptual qualities. In disorders of consciousness, such aspects of consciousness are disrupted in a way that negatively affects the subject’s well-being.

Although we are all intimately familiar with consciousness from a subjective, first-person perspective, the scientific study of consciousness is less definitive [29], [30], and many empirical theories of consciousness make competing claims [31]. Still, our theoretical and empirical understanding of consciousness has improved significantly during the past decades [35], [36], [32], [33], [34]. With its close link to computational neuroscience, CP can benefit from such progress through translational neuromodelling. This highlights the potential of CP in deepening the understanding and improving the treatment of disorders of consciousness (in the general sense).

Successful applications of CP may thus reshape how we conceive of disorders of consciousness and thereby also affect our understanding of ‘normal’ conscious experiences. Changing our conception of normal and disordered conscious experiences might reduce or reinforce stigma and increase or limit treatment options. The potential transformative effects of CP are therefore morally relevant. In particular, they may reduce or increase the well-being of persons suffering from mental disorders. For this reason, they are also relevant from the point of view of an ethics of consciousness [39], [37], [38].

In this paper, we discuss potential transformative effects on the concept of mental illness that successful clinical applications of CP may have—even if researchers do not intentionally pursue the goal of revising existing (symptom-based) diagnostic categories. In particular, we address the worry that this type of research might reinforce a biological reductionist view of mental disorders [40], at the risk of ignoring sociocultural factors and changes in conscious experience associated with mental disorders [20].

The paper is structured as follows. First, we provide a succinct description of computational psychiatry and ethical issues in AI. After that, we explore some prospects and pitfalls of CP in the following three steps:

● **Thesis**: Computational psychiatry is tendentious; many branches of CP have a focus on brain function. This may reinforce a biological reductionism, ignoring psychosocial aspects of mental disorders.
● **Antithesis**: Computational psychiatry is neutral with respect to metaphysical questions about mental disorders; computational models can take all aspects of mental disorders into account and therefore need not presuppose a definition of mental disorder that only considers, say, biological variables.
● **Synthesis**: Computational models can increase our understanding of psychosocial aspects; but computational models can also fail to take them into account. Hence, to maximise its benefits, CP should be aware of the risk of *unintentionally* marginalising subjective experience. Otherwise, the potential impact of research in CP on clinical practice may fail to be fully realised or may even be partially detrimental.

We illustrate the remaining worry in the synthesis with a case study. We conclude that CP can and should—at least in the long run—provide a better understanding of what a ‘good,’ ‘normal,’ and ‘pathological’ conscious experience is. The ethics of computational psychiatry will then also be an ethics of consciousness [39], [37], [38].

## 2 What is computational psychiatry?

Computational psychiatry (CP) [5], [6], [2], [4] seeks to translate findings—and techniques—from computational neuroscience to clinical psychiatry [41], in order to enable a deeper understanding of mental disorders [42], to improve diagnostics, to enable precise and reliable prognostics and therapy prediction and, ideally, to develop new therapeutic approaches. Apart from these, a long-term goal of CP is to improve diagnostic categories by leveraging, nuancing or replacing symptom-based nosologies [1], [3], [7], [19].

A key assumption within CP is that computational models can be used to define computational (endo)phenotypes [43]. Ideally, this will not only provide valid characterisations of mental health and illness, but also a bridge between molecular and behavioural findings [4]. In the long run, this can enable precise, mechanistically grounded and effective therapeutic interventions and thereby improve outcomes for patients [44].

It is common to distinguish between two branches (or ‘cultures,’ [45]) of computational psychiatry: data-driven and theory-driven approaches [46], [44].^1^ Data-driven approaches use machine learning to analyse and label data. This can enable classifications and predictions of—among others—treatment responses [47] or the trajectories of mental disorders (e.g., of major depressive disorder, [48]). Apart from some exceptions, theory-driven approaches use *generative models* to model the causes of data. In contrast to discriminative models (which can only be used to classify data and their likely causes), generative models embody hypotheses about how observed outcomes have been generated; this also enables simulations and evidence-based comparison between hypotheses, through Bayesian model selection [49].

A generative model is a probabilistic model of (observable) data and their hidden (unobservable) causes. Such models or hypotheses can be used to infer the underlying mechanisms of symptoms, behavioural signs, or measurements (e.g., obtained using fMRI, [51], [52]) or, indeed, conventional symptom-based diagnoses [1]. Ideally, this can facilitate differential diagnoses for individual patients; in this context, generative models are also called *computational assays* [7]. If successful, they could allow for more precise and reliable diagnoses and therapy predictions, which is already a morally praiseworthy aim (provided the same effects cannot be brought about in a less expensive or less time-consuming way). In addition to this, computational assays promise to improve purely data-driven approaches. For instance, computational assays may improve machine-learning-based stratification (i.e., clustering into specific subgroups) by *generative embedding* [55], [53], [54] in at least two ways. First, generative embedding reduces the dimensionality of data by fitting a generative model with interpretable parameters; this allows representing data from subjects by a small number of features, which can also improve the performance of algorithms. Second, this can provide information about *why* patients are divided into certain subgroups by a machine learning algorithm, because the features used by the algorithm are mechanistically interpretable [56], [54].

In contrast to approaches using generative models, data-driven approaches need not make their assumptions explicit in the form of a generative model. To a certain extent, this means one can let the “data […] ‘speak for themselves’” ([57], p. 223). However, this does not mean that decisions made by researchers do not affect the outcomes of data analysis and prediction. On the contrary, specific care has to be taken, in order to avoid outcomes that are biased or do not generalise, due to decisions regarding, e.g., data collection and data pre-processing ([58], p. 72). In particular, this means that applications should be validated in independent samples.

Model parameters in machine learning are not usually interpretable. In spite of this, even ‘black-box’ algorithms can have high predictive accuracy ([59], p. 254). Such methods can therefore still be highly useful for various purposes in psychiatry, for instance, for predicting future alcohol misuse or suicidality [61], [60].

Nevertheless, non-interpretable (data-driven) approaches can be problematic when errors occur, and patients are harmed. This brings us to ethical problems in computational psychiatry.

## 3 AI ethics and computational psychiatry

The goals of CP have direct ethical implications, due to their potential to serve patient well-being and because of the risks involved. Most ethical issues associated with CP’s main goals are already known, in similar form, in biomedical ethics [62], neuroethics [64], [63], and AI ethics [65]. Examples include the handling of incidental findings [66], the possibility of improved early detection of disease risks [67], consequences for our self-image as autonomous, self-effective agents [68], or problems of data protection [69] and algorithmic biases [18]. For a discussion of such problems in the context of computational psychiatry, see [13], [15].

Such problems should not conceal the potential benefits of CP. Mental illnesses are globally among the leading causes of disability-adjusted life years (i.e., years lived with disability plus years of life lost, [70]). At the same time, access to mental health care is often severely restricted, both in low-income countries and high-income countries [71]. For instance, in 2015 a study found that the median duration of untreated psychosis in community clinics in the US was 74 weeks [72]. This shows that mental illness itself is a global morally relevant problem because it goes along with suffering and is in most cases not adequately treated. Refining diagnostics and treatments, in order to improve patient outcomes, is therefore a morally praiseworthy goal.

The ethical issues associated with applications of AI in psychiatry, and of CP in particular, can more systematically be described by distinguishing the different domains of applications (e.g., early detection, diagnosis, and treatment, see [40]) and by reference to (biomedical) ethical principles, such as beneficence, non-maleficence, respect for autonomy, and justice [62]. For AI applications, there is a further fundamental principle: explicability [12], also called *transparency* or *explainability*, which has a normative and an epistemic aspect. An AI application is explicable in the epistemic sense if it is intelligible how the system works, e.g., if it is transparent why it classifies a given input in a particular way. It is explicable in the normative sense if one can determine who is responsible for the way the system works and who is accountable for its outcomes. This is especially relevant when an application fails to work in the intended way or if it has undesirable consequences. Examples include applications with racist or other biases [73].

The project of developing *computational assays* is especially interesting from an ethical point of view, because it can lead to interpretable results (see above). More generally, certain projects within theory-driven CP (as opposed to purely data-driven CP) promise to enable *explainable* applications, thereby circumventing the black-box problem known from AI ethics [74].

Apart from the huge potential benefits of CP, there is the concern that most approaches in CP are too narrow, in that they tend to focus on biological properties and fail to take psychosocial factors into due consideration [40]. In particular, one might worry that CP shares problems of the ‘third wave of biological psychiatry’ [75], according to which mental disorders are either brain disorders or can be diagnosed and treated without paying much attention to psychosocial factors. This, however, would mean that central aspects of mental disorders are ignored [20], thereby leading to suboptimal treatments (at least potentially); in particular, this cannot do justice to disorders of consciousness.

These considerations make CP particularly interesting from the point of view of an ethics of consciousness [39], [37], [38]. On the one hand, CP bears the prospect of alleviating suffering, which, in most cases, is morally praiseworthy. On the other hand, it bears the risk of ignoring, and failing to treat, crucial aspects of disordered conscious experience, which would be morally blameworthy.

Taking AI ethics as a starting point may be especially useful in this context because there can be a tendency to think that a purely technical solution will be found [76], or that thorny problems such as unfairness of AI applications can be fixed by achieving complete AI fairness [77]. Similarly, it addresses the specious belief that any improvement of CP applications will dissolve or mitigate any ethical concerns. Drawing on insights from the more general debate on AI ethics could therefore help avoid similar problems or misconceptions in the context of CP.

In the remainder of this paper, we probe the concern that CP might promote tendentious views of mental disorders, thereby impeding efforts to realise CP’s full potential. After considering arguments in support of this concern, as well as counter-arguments, we try to do justice to both side of the debate, by distiling the key aspects of the concern that remain, even after considering objections. The central remaining worry is that even successful applications of CP can, in the long run, fail to adequately treat all aspects of disordered conscious experience. This concern should not be regarded as an objection to approaches in CP, but as a chance to maximise the benefits of CP: computational approaches have the required resources and should therefore be leveraged to account for even subtle and puzzling aspects of (disordered) conscious experience.

## 4 Thesis: Computational psychiatry is metaphysically tendentious

Superficially, it may seem that CP does not presuppose any assumption about the nature of mental disorders. In particular, CP is not committed to the claim that mental disorders are brain disorders. For instance, Adams et al. [49] stress that “Computational Psychiatry […] can unite many levels of description in a mechanistic and rigorous fashion, while avoiding biological reductionism and artificial categorisation.” ([49], p. 53). In a similar vein, Huys et al. [44] assert:

“[W]e emphasise that illnesses are complex phenomena defying simplistic aetiological or mechanistic accounts […]. Indeed, research has identified contributions to the syndromes we identify as disorders arising at different levels from genetics to neural circuits, psychological processes, and social or societal factors. From a broad computational view, all of these factors lead to a mismatch between the brain’s computational ability, and the environmental or situational demands placed upon it.” ([44], p. 3).

This highlights the fact that computational models are, in principle, metaphysically neutral. In particular, computational models need not focus on neural data, but can also take subjective reports and even social interactions into account ([78], p. 549). This suggests that it is at least an open question whether a future nosology, based on successful clinical applications of CP, will construe mental disorders as, for instance, disorders of the brain [79], as mechanistic property clusters [80], or, more specifically, as symptom networks [81], [82].

In practice, however, many approaches in CP tend to focus on brain function ([2], p. 148; [4], pp. 72–73; [5], p. 22; [7], p. 85). In an influential landmark paper, Montague et al. [4] claim:

“[T]he brain is the organ that generates, sustains and supports mental function, and **modern psychiatry seeks the biological basis of mental illnesses**. This approach has been a primary driver behind the development of generations of anti-psychotic, anti-depressant, and anti-anxiety drugs that enjoy widespread clinical use. Despite this progress, biological psychiatry and neuroscience face an enormous explanatory gap. […] We believe that advances in human neuroscience can bridge parts of the explanatory gap. […] It is the computational revolution in cognitive neuroscience that underpins this opportunity and argues strongly for the application of computational approaches to psychiatry.” ([4], pp. 72–73, bold emphasis added).

If CP merely contributes to understanding how neural processes can be changed using drugs,^2^ then it is to be expected that such research will reinforce the view that mental disorders are disorders of the brain. This focus is too narrow, for at least four reasons.

First, the concept of a mental disorder is a normative concept. Of course, certain types of neural activity can also be regarded as aberrant forms of information processing (e.g., as inferences based on suboptimal models, [83])—i.e., CP itself often uses normative concepts. But the norms of optimal information processing and the norms of mental health can diverge ([84], p. 453). For instance, social anxiety *reduces* positive self-referential bias [85], [86]. Hence, mental disorders cannot simply be identified with suboptimal information processing.

Second, disorders that involve mental states with illusory or false contents (e.g., hallucinations or paranoid beliefs) essentially depend on the subject’s environment: whether a belief is true or false, for instance, cannot be determined by looking at the subject’s brain. Borsboom et al. provide the following example:

“Elizabeth and Bob may both believe that they are persecuted by the CIA, and this belief may be instantiated in the exact same way in their brains. Depending on the external circumstances, however, this belief may count as a symptom or not – for instance, when the belief is veridical for Elizabeth (who is actually a Russian spy) but finds no grounding in reality for Bob.” ([87], p. 49).

Even assuming that the content of a belief can be understood in terms of neural properties, it does not follow that a model of neural mechanisms allows one to determine whether the belief is true or false, which would be required to distinguish pathological from non-pathological beliefs or inference.

Third, as emphasised by 4E approaches [88], many mental states are embodied, embedded, enactive, and extended. Therefore, it is unlikely that mental phenomena (whether pathological or healthy) can be identified with neural states and processes [75].

Fourth, even if conscious experience is an exception to the former point and can be reductively explained in terms of neural properties, there is the risk that applications of CP will ignore consciousness and lead to a “Zombie-psychology” [89]. As Huys et al. put it: “People pay for psychiatric help partly because internal subjective experiences have external objective correlates: because they cannot work or look after their children, not just because they feel sad” ([78], p. 545). Zombie-psychology may take care of external objective correlates and help patients become ‘functional’ again (which is, of course, fine), but disorders of conscious experience may persist.

To the extent that CP focuses on the brain, it therefore presupposes problematic assumptions about mental disorders. These assumptions may reinforce overly narrow conceptions of mental disorders, limit treatment options, and increase stigmata associated with mental disorders [90]. This can decrease the probability that affected persons will seek help [68].

Instead of regarding mental disorders as brain disorders, it has been theoretically fruitful to regard mental disorders as mechanistic property clusters (MPCs) [80]. MPCs are clusters of causal mechanisms that can interact and mutually sustain one another. Crucially, mental disorders typically involve many different causal mechanisms and mental disorders are multiply realisable ([80], p. 1148); this precludes mono-causal explanations of mental disorders [91].

A specific version of the MPC view is the symptom-network approach [81], [82]. Here, the idea is not to define mental disorders in terms of clusters of underlying *causes* of symptoms, but as causal networks of *symptoms*. The approach starts from the following assumptions: “(1) mental disorders are massively multifactorial in their causal background; (2) many mechanisms that sustain disorders are transdiagnostic; and (3) mental disorders require pluralist explanatory accounts” ([82], p. 3)In particular, the network approach assumes that, once symptoms have been activated (due to external conditions or internal dysfunction), they can cause other symptoms (for instance, insomnia may cause fatigue) and may stabilise one another, even when the external cause is no longer present ([82], p. 4). Furthermore, the way symptoms interact often depends on sociocultural context ([82], pp. 7–8). Defining mental disorders as symptom networks therefore offers the chance “to integrate the biological, psychological, behavioural, and environmental mechanisms that create causal relations between symptoms” ([82], p. 11).

CP, on the other hand, has at least a tendency to ignore psychological and environmental mechanisms. It thereby misses the chance (offered by the network approach) to integrate multiple relevant factors, which would lead to a comprehensive understanding of mental disorders.

## 5 Antithesis: Computational psychiatry is metaphysically neutral

It is correct that some approaches within computational psychiatry focus on neural mechanisms. However, this does not mean that computational psychiatry is tendentious or that it is committed to ignoring psychosocial factors. In fact, many applications of machine learning in psychiatry include a diverse set of data in their analysis. Let us just give two examples to illustrate this point.

In a longitudinal study with a large sample of adolescents, Whelan et al. [61] investigated factors that can be used to predict current and future alcohol abuse. Crucially, the data reflected “brain structure and function, individual personality and cognitive differences, environmental factors (including gestational cigarette and alcohol exposure), life experiences, and candidate genes” ([61], p. 185). Such approaches are therefore not committed to a narrow focus on a particular type of data (e.g., neural data).

A more recent study by Koutsouleris et al. [60] used machine learning to predict psychosis in patients with clinical high-risk states. The data included clinical-neurocognitive, genetic, and structural imaging data. It turned out that risk predictors based on clinical-neurocognitive data could explain most of the variance in the sample, followed by predictors based on genetic and structural imaging data. Since data from clinical interviews include information about psychosocial factors, the data considered were quite comprehensive. What is more, this study also illustrates an advantage of data-driven approaches: rather than deciding a priori which variables should be taken into account, such approaches provide a rigorous way of testing to what extent the different factors are relevant.

Although these are just two examples, it should be obvious that a commitment to computational methods does not entail a commitment to using only certain types of data. By contrast, data-driven approaches are flexible enough so as to consider diverse data sets, thereby “allowing the data to ‘speak for themselves’” ([57], p. 223).

Similarly, approaches using generative models can take interactions between the brain and external processes into consideration. Smith et al. [92] provide a compelling illustration of how this can be used to integrate and extend models of major depressive disorder. Far from identifying mental illness with ‘pathological’ biological mechanisms, their model construes major depression as arising from nested feedback loops spanning brain, body, and the social environment. Because of the comprehensive nature of this approach, it not only enables hypotheses about the aetiology and heterogeneity of major depressive disorder, but also regarding pharmacological and psychotherapeutic treatment mechanisms.

Let us now address the more specific concerns raised above. Recall that these concerns refer to (1) the normativity of mental health and pathology, (2) the role of the environment, (3) the relevance of 4E approaches to understanding mental disorders, and (4) the risk of neglecting conscious experience.

(1) It is correct that the norms of mental health cannot be identified with the norms of optimal information processing—in particular, because some disorders reduce certain biases [84]. But such ‘optimisation’ will go along with other disadvantages, which can, e.g., be understood in terms of suboptimal models [83]. These can be ‘suboptimal’ due to changes in the model parameters of the (generative) models patients use to make sense of their world [93]. Furthermore, at least some (statistical) norms of mental health can be clarified with *normative models* that quantify the extent to which individuals deviate from a statistical norm [94].

The deeper point seems to be that computational psychiatry cannot disentangle itself from existing diagnostic categories and norms, but must embrace them. To the extent that approaches in CP deny this point, they are doomed to fail.

Although important, this point overlooks the fact that work in CP can build on existing categories (with their implicit norms), without reifying them. For instance, an important goal is to enable more fine-grained diagnoses by dissecting spectrum disorders ([95], p. 727). A more radical and straightforward approach is to consider existing categories as the product of a measurement process—and test generative models of how these diagnostic measurements were generated in terms of pathophysiology and psychopathology [1]. Moreover, CP promises to improve prevention, prognoses, and treatment predictions, but none of these goals requires ignoring existing categories and norms. Still, CP offers the potential to *improve* diagnostic categories—which, almost by definition, is ethically desirable. The same holds for improving treatments and predictions.

(2) Hallucinations or delusional beliefs cannot be understood exhaustively in terms of neural properties: the veracity of many beliefs depends on the environment. However, the deeper source of suffering is not the lack of veracity of a particular belief or hallucination, but the tendency to form such pathological mental states in the first place. In fact, one could even argue that not individual beliefs, but rather the ways in which beliefs are formed and updated (a.k.a. inference), can be regarded as pathological. Although the difference between a sincerely-held false belief and a true belief is not something that can be captured by a model of neural processes, the internal dysfunction leading to a failure of adjusting one’s beliefs *can* be modelled in this way. More specifically, hallucinations and delusions can be modelled in terms of aberrant belief-updating [24], [93], [96]. These positive symptoms of psychosis correspond to a particular type of false inference: inferring something is there when it is not. The complementary second type of false inference is inferring that something is not there when it is (e.g., various neglect and agnosia syndromes).

(3) The third concern emphasises that mental states are embodied, embedded, enactive, and extended [88]. This suggests that mental phenomena (whether pathological or healthy) cannot be identified with neural states and processes [75], but it does not mean that understanding brain function is irrelevant to understanding mental states. For instance, Miller et al. [97] draw on computational models to develop an ecological-enactive account of addiction. Although the authors take computational models of how addiction affects midbrain dopaminergic systems into account, they do not construe addiction as a brain disease. Instead, they argue that addiction should be regarded as an embodied phenomenon, which is ultimately not simply a disease of the brain, but a problem of living. This shows that computational approaches in psychiatry leave room for interpretation and do not presuppose contentious metaphysical assumptions about the nature of mental disorders.

(4) The charge that CP runs the risk of ignoring subjective experience can easily be dispelled. Disorders of consciousness are among the symptoms of many mental disorders, e.g., hallucinations in psychosis [24], or deviant time- and self-consciousness in major depressive disorder [25], depersonalisation disorder [26], and schizophrenia [27]. The relevance of computational approaches to understanding aspects of disturbed consciousness has already been demonstrated (for a few examples, see [24], [98], [99], [100], [101], [102], [103], [104], [105], [106], [107], [108], [109], [110]). Hence, it is not the case that CP must ignore, or cannot be applied to, disorders of conscious experiences.

On the contrary, CP has the potential to improve existing diagnostic categories for disorders of consciousness, by incorporating correlates of consciousness. As Henrik Walter puts it:

“Because every mental state has a correlate in the brain, we should be able to find at least in principle neurobiological correlates of any mental state, pathological or not. So the question is not, whether there is a neurocognitive correlate or mechanism, but whether it is pathological, how it came into being, whether it is persistent, whether and how it can be influenced, and so forth.” ([75], p. 5; see also [111], p. 86).

Findings about neural correlates of mental states provide further data that can inform diagnostics, prognostics, treatment decisions, and nosology. This does not presuppose that neural correlates reveal everything there is to know about a condition. In particular, a neural correlate itself does not tell us whether the accompanying mental state is pathological or not. It does not replace subjective assessments of well-being. However, this—in and of itself—does not preclude leveraging neuro-computational findings in a therapeutic setting.

This suggests that a focus on brain function is, in itself, metaphysically neutral. For the relevant question is not whether research in CP focuses on brain function, or also takes psychosocial factors into account. The relevant question is how findings about neural and computational correlates of pathological mental states and symptoms inform the way mental disorders are categorised and classified. Even if, for instance, a computational model is used to infer the neuronal mechanisms underlying pathological symptoms, it is possible to regard neuronal mechanisms as just one factor among many that jointly constitute or cause the observed symptoms ([112], p. 35).

This also speaks to the notion of mental disorders as mechanistic property clusters [80]—or, in particular, as symptom networks [81], [82]. Such approaches may have the potential to integrate multiple relevant factors and foster a comprehensive understanding of mental disorders, but one can make the case that they should be complemented by computational modelling ([112], p. 36).

For instance, although correlations between symptoms and signs can be revealing, it will ultimately be expedient to investigate causal relations between the mechanism underlying measurements (including subjective reports). Friston et al. [1] illustrate this point as follows:

“[T]here is a fundamental distinction between a measurement (e.g., a temperature of 38.2 °C) and the causes of that measurement (e.g., bacterial infection). It is almost self-evident that to generate the (profile of) measurements available to a clinician, it is necessary to model their latent causes, whether or not they are ontologically well-defined. […] [W]e should try to identify the causal (network) architecture among the symptoms’ latent causes: namely, the best generative model. Both symptom network analysis [113] and generative modelling eschew the common-cause framework—namely, the assumption that symptoms and signs can be uniquely attributed to a common cause.” ([1], p. 19).

In addition to making a distinction between measurements and their causes, it may also be necessary (and illuminating) to make a distinction between data and symptoms. As Fellowes [114] argues in the context of autism spectrum disorder, symptoms must be inferred on the basis of data and may even be, in some sense, constructed. In the network approach, this becomes manifest in the fact that network analyses will yield different results, depending on whether they are conducted on the basis of a DSM/ICD taxonomy, or, for instance, on the basis of the Research Domain Criteria ([112], p. 36).

Furthermore, computational modelling approaches can be useful for understanding correlations between different types of symptoms. For instance, there is a correlation between psychiatric disorders and immune responses [115]. Bhat et al. [116] show how hypotheses about the nature of this connection can be computationally modelled and explored through simulations *in silico*. There are thus many ways in which CP can (and should) augment network approaches.

More generally, CP can furnish a mechanistic understanding of relationships between psychological, biological, and social variables. As Smith et al. [117] show with respect to health and social support, neurocomputational approaches can go beyond investigating correlations, by formulating and exploring implications of testable hypotheses about how such variables are causally related. In particular, this also involves investigating how biological, psychological, and social processes are regulated within individual brains. Hence, as the authors point out, there is a sense in which “all the major elements of the biopsychosocial model are […] *already present* within any complete biomedical model” ([117], p. 141).

Since the focus of this paper is on disorders of consciousness, it should be noted that it can sometimes be useful, or even necessary, to ignore some aspects of conscious experience. Consider the problem of predicting the risk for suicidal behaviour. Data for predictors of suicidality typically include subjective reports of suicidal ideation, because suicidal ideation has for a long time been regarded as a central index for suicidality [118]. However, suicide attempts need not be preceded by suicidal ideation [119], and some persons may be unwilling to report suicidal ideation. For these reasons, it is especially useful that computational approaches can be used to predict suicidal behaviour without having to rely on reports of suicidal ideation [120].^3^ In this case, ignoring conscious thoughts (suicidal ideation) is not just acceptable, but even desirable, because it can help make predictors more accurate.

## 6 Synthesis

In this section, we take stock, by highlighting CP’s potential for progress, while acknowledging remaining concerns. We restrict the discussion to the transformative potential of CP, focusing in particular on the prospects of an improved nosology.

One line of argument—presented in the thesis above—has it that many CP approaches focus on brain function, which is ultimately too narrow to be successful. In the antithesis, this argument was countered by pointing out that (i) many approaches in CP are much broader and (ii) it can often be useful to restrict the focus (without presupposing that mental disorders are brain disorders). Thus, there is currently no reason to believe that CP will not be able to realise its potential because of an alleged narrow focus. Still, one should beware of tendencies to view computational approaches merely as a means of developing more effective pharmacotherapy. That is, it should be acknowledged that successful applications of CP may improve diverse types of therapeutic approaches and foster a comprehensive understanding of mental illness; but individual results could be instrumentalised to pursue a more narrow-minded agenda—for instance, Starke et al. [15] raise the worry that “lobbying by pharmaceutical companies might have an interest to split psychiatric disorders into many distinct categories to gain advantages in the approval of new drugs.”

### 6.1 Transformative effects of computational psychiatry

One can add that even models with a restricted focus need not affect views about the nature of mental illness. For instance, even if hallucinations and delusions are modelled as aberrant belief-updating [24], [96], this does not mean that the brain literally processes information in this way. Instead, one can interpret such models instrumentally, suggesting that it can be useful to view (some) mental disorders or symptoms in this way, without presupposing that mental disorders are brain disorders. In particular, models in CP need not claim to provide the only way in which a mental disorder can be conceived. Nevertheless, such models can have profound effects. For instance, Colombo and Fabry suggest they may “re-shape people’s image of delusion, and possibly impact the nature of delusional experience itself.” ([98], p. 22). Changing people’s images of delusion and other symptoms can be beneficial (e.g., if it provides a way of coping with a condition), but it could also have harmful effects (e.g., by increasing stigmata).

A remaining worry is that successful applications of CP might still fail to fully take psychosocial factors into account. The worry is not that this will happen intentionally, or because CP has an inherent tendency to ignore such factors—the rebuttals in the antithesis should have clarified that work in CP can and often does incorporate data on psychosocial factors.

Still, the risk that CP may fail to properly address disorders of conscious experience should be taken seriously. Note that the problem is not that CP lacks the concepts or methods to take features of disordered conscious experiences into account. As indicated above, existing work speaks against this suspicion [24], [98], [99], [100], [101], [102], [103], [104], [105], [106], [107], [108], [109], [110]. Rather, the problem is that some features of scientific progress may drive CP into a trajectory that converges on diagnostic criteria that fail to include at least some aspects of disordered consciousness.

In what follows, we shall describe two ways in which CP might, in the long run, lead to a revision of diagnostic categories that ignores important features of disorders of consciousness. The two possibilities described are to some extent speculative, but should be taken into consideration as part of an ‘ethical risk assessment’ of computational psychiatry. We do not believe that the potential harms entailed by these risks outweigh the potential benefits of successful applications of CP. However, we do believe that being mindful of these risks can help maximise the benefits of CP.

### 6.2 Why should the risk of discounting consciousness be taken seriously?

The first reason why crucial aspects of disorders of consciousness might end up being ignored is methodological. Developing and validating generative models for spectrum disorders such as schizophrenia is a complex task, requiring longitudinal studies with many participants. Focusing on biological features reduces the complexity of the task, without simplifying it: even if a complete understanding of a mental disorder also requires taking psychosocial factors into account, biological approaches can paint an important part of the picture. As Huys et al. put it: “From a broad computational view, all of these factors lead to a mismatch between the brain’s computational ability, and the environmental or situational demands placed upon it.”([44], p. 3). The mismatch will not be understood without considering the environmental or situational context, but at least the brain’s computational ability can ideally be assessed by narrow (biological) approaches in CP. Apart from this, there can be economic incentives to focus on biological variables and the development of medical interventions (in particular, therapeutic drugs).

Incidentally, even George Engel, the pioneer of the biopsychosocial model, suggested that excluding certain aspects of mental illness can be reasonable [121]. However, he added that this exclusion can become problematic in the long run: “[I]t becomes counterproductive when such strategy becomes policy and the area originally put aside for practical reasons is permanently excluded, if not forgotten altogether. The greater the success of the narrow approach the more likely is this to happen.” ([121], p. 131). To what extent can it be counterproductive to adopt a narrow approach in CP, and how can it be problematic, if it is successful? The answer is that success can be partial. For instance, a narrow approach may improve prognoses and treatment predictions for some symptoms (e.g., in severe cases). This could count as a success and could motivate efforts to refine existing treatments and further improve predictions, without even addressing other symptoms (which may be less severe or more difficult to assess). In the long run, some aspects of a subject’s ailments will be cured, but other aspects that are harder to measure may persist. Below, we illustrate this point with a case study.

The second way in which biological approaches within CP may fail to take features of disturbed conscious experiences into account is motivated by a suggestion in the antithesis, according to which even a focus on brain function can be regarded as metaphysically neutral. Conscious experiences—whether pathological or not—have neural correlates ([75], p. 5); biological approaches in CP can complement research on neural correlates by shedding some light on computational correlates of consciousness [122]. Furthermore, there may be characteristic cognitive or behavioural correlates. Investigating such correlations can advance the understanding of mental disorders.

A potential problem is that some of these correlates may receive more attention than the features of consciousness with which they correlate—because behavioural and biological variables can be easier to measure and may enable a more reliable categorisation. Of course, reliable diagnostic categories are desirable, but this should not be at the cost of other relevant variables.

Crucially, a shift to behavioural and biological factors, at the neglect of psychological factors, can be motivated by initiatives like the RDoC approach [123]. Although RDoC’s units of analysis include self-reports as one unit among seven, there is a clear emphasis on biological indicators [124], [125]. This can create the impression that biological factors are more fundamental [126]. Furthermore, the RDoC initiative explicitly fosters attempts to discount subjective reports of psychological problems, by replacing them with data that speak to the mechanisms underlying the reported psychopathology: “Ultimately, if the RDoC initiative proves successful, psychobiological mechanisms might usurp the telltale role of self-reported experiences in a renovated diagnostic system.” ([127], p. 933). A potential risk is that even successful applications may target only *some* of the underlying mechanisms, thereby leaving important aspects of some mental disorders unaddressed. To prevent this, considering subjective reports remains indispensable. For it would be premature to expect that computational methods will reveal the neural or computational underpinnings of subjective well-being. That is, when it comes to evaluating to what extent not only individual symptoms, but also a patient’s overall condition has improved, the primary authority remains the patient themself.

This is not just a potential problem of the RDoC approach, but of progress in psychiatry more generally. Subjective reports can be ambiguous and unreliable. For this reason, there will always be some pressure to refine methods that tap into the ‘objective’ factors of mental disorders. To the extent that such efforts are successful, the subjective factors of mental disorders can be discounted, because they have already been accounted for in terms of other, more reliable variables—or so one might argue.

### 6.3 Potential side effects of progress in psychiatry

We shall now consider this possible dynamics of progress in psychiatric research in a bit more detail. To this end, it will be instructive to see how progress in other disciplines has replaced subjective measures with more reliable, objective measures. In particular, we shall see that this is a potential outcome of what Chang [128] calls *epistemic iteration* (see also [129]). After briefly introducing this concept, we shall review a recent application to psychiatry by Colombo [130], who applies the concept to research on alcohol use disorder. Afterwards, we show how the concept can be applied to research on schizophrenia (see also [131]), and suggest that a potential side effect of epistemic iteration is the neglect of certain aspects of conscious experience.

Chang defines the notion of epistemic iteration as follows: “Epistemic iteration is a process in which successive stages of knowledge, each building on the preceding one, are created in order to enhance the achievement of certain epistemic goals.” ([128], p. 45). Epistemic iteration is thus a particular type of scientific progress, and it need not involve theory falsification, as in Popper’s hypothetico-deductive model [132], nor scientific revolutions in the sense of Kuhn [133]. Chang develops this concept in the context of the history of thermometry, i.e., the measurement of temperature.

Even before thermometers were invented, temperature could be measured—albeit imprecisely and non-reliably—by bodily sensations. More reliable measurements could be obtained using devices containing fluids that expanded when they were heated. Following Middleton [134], Chang [128] calls such devices *thermoscopes*. In contrast to quantitative thermometers, the former only have ordinal scales. Developing thermometers with cardinal scales requires fixed points, such as the freezing and boiling of water. Once fixed points were found (which is non-trivially, without already having a reliable thermometer), numerical thermometers could be created, which enabled quantitative measurements of temperature. This also enabled theoretical advances:

“By means of numerical thermometers, meaningful calculations involving temperature and heat could be made and thermometric observations became possible subjects for mathematical theorising. Where such theorising was successful, that constituted another source of validation for the new numerical thermometric standard.” ([128], p. 48).

However, epistemic iteration does not stop here. The boiling point was only one candidate for a fixed point; a competing candidate was the “steam point,” and the latter eventually replaced the boiling point, because it was more stable and was supported by theory ([128], p. 48). Further iterative refinements and extensions of thermometry took place, involving an interplay between theoretical and empirical advances.

Colombo [128] applies this concept to the role reinforcement learning plays in the study of alcohol use disorder. As the starting point for the scientific study of alcohol use disorder, Colombo identifies phenomenological observations, clinical experience, and patients’ needs. Based on these, correlations between phenomenological observations, risk factors, environmental cues, and behavioural and biological symptoms can be investigated using empirical studies. “Fixed points” are “implicitly or explicitly employed—such as, for example, a definition of substance use disorders grounded in heavy use over time [135]—for triangulation and probing the reliability and validity of these correlations” ([130], p. 15). Colombo notes that computational explanations in psychiatry can take different forms, including aetiological and constitutive explanations.

We can apply this to the scientific study of schizophrenia. For the purpose of this paper, the starting point can be identified with a definition of schizophrenia in terms of a set of positive and negative symptoms and signs (as in DSM-V). This brushes over many historical complexities (e.g., the ‘neo-Kraepelinian revolution’ constituted by drastic changes in the transition from DSM-II to DSM-III, see [136]). However, our focus is on how epistemic iteration may play out in the future, not on how it may have been at work in the past.

“Fixed points” for the study of schizophrenia are given by particular types of symptoms (e.g., auditory hallucinations or delusions). Since schizophrenia is a spectrum disorder, these fixed points are far from perfect, but nevertheless useful as starting points. Moreover, this also illustrates why iterative refinements (in the sense of epistemic iteration) are particularly useful in the context of schizophrenia. Empirical studies reveal correlations between behavioural and neurophysiological variables. Computational models can be used to explore hypotheses and derive predictions, which can be tested by adapting the cognitive tasks used in empirical studies. Crucially, this is where iteration occurs, assigning a central role to computational modelling. Deserno et al. characterise this process as follows (in the context of negative symptoms of schizophrenia): “cognitive tasks studying reinforcement learning and decision-making have been shown to be associated with negative symptom severity with at least some consistency. This can be improved by mutually refining learning and decision-making tasks and computational models” ([137], p. 52).

Of course, we can only speculate what concrete results this process of epistemic iteration will yield in the near future. However, we can make a guess that is consistent with some aims of computational psychiatry. A mid-term result may be that machine learning is used to make a personalised treatment prediction for individual patients. As an illustration, consider the following hypothetical example by Starke et al. (involving a fictional patient ‘T’):

“T is **diagnosed with a first episode of schizophrenia based on a clinical interview**. To choose the most effective drug for his individual situation, his psychiatrist recommends a newly approved routine employing functional MRI during a reward-learning task. Based on T’s brain activity and a plethora of other available information, from demographic data to his clinical records, the **ML algorithm suggests one specific anti-psychotic drug** as ideal for T’s specific situation. Following the automated recommendation, the psychiatrist prescribes the drug to her patient.” ([15], p. 3, bold emphasis added).

In this case, machine learning is used for personalised treatment prediction, but the diagnosis is still based on a clinical interview. As a long-term result, we can imagine that the entire clinical interview [138], or at least the diagnosis resulting from it, will be treated as a yet another data point. Together with further measurements, it is fed to an algorithm, or informs the choice of a generative model, which is then used to infer the underlying mechanisms, on the basis of which the final diagnosis is made [1]. The diagnostic categories used will be more fine-grained than the ones used in DSM-V (and ICD-10), while at the same time ignoring the categorical boundaries between disorders implied by neo-Kraepelinian nosologies. This enables not just a more reliable and precise diagnosis, but also more accurate treatment predictions.

At the same time, subjective reports may become less relevant to diagnoses, in line with goals of the RDoC approach ([127], p. 933). Just as the development of quantitative thermometers rendered subjective sensations of warmth and cold superfluous as measures of temperature (at least for scientific and diagnostic purposes), a reliable blood test [139] or computational assays [55] for schizophrenia might make subjective reports more or less dispensable.

The analogy with thermometry illustrates how CP can successfully promote progress in research on schizophrenia that results in tangible applications. At the same time, it highlights the risk of neglecting aspects of disordered conscious experience. For there is also a key disanalogy: thermometry was never meant to yield an understanding of *subjective* sensations of heat; it was meant to yield reliable, quantitative measurements of properties of external objects and processes. In the case of disorders of consciousness, this is different. There is thus always the possibility that objective measurements fail to capture all aspects of the disorder to which subjective reports point.

### 6.4 A case study involving disturbed temporal experience

In order to make this more concrete, consider the following case study by Martin et al. [140]. The case study shows that successful treatments can be partial: even if some symptoms of a condition have disappeared or are alleviated, other—perhaps more subtle—symptoms may persist. What is more, these residual symptoms need not be negligible, but can instead constitute a significant disruption of conscious experience. This illustrates that applications of CP might succeed in, for instance, developing effective personalised treatment recommendations, while at the same time failing to improve patient outcomes in other crucial respects. Martin et al. [140] cite reports by a young man, AF, who had previously been diagnosed with schizophrenia:

“When we encountered AF, functional impairments persisted, i.e., difficulty in social and professional integration, but there was no longer any obvious behavioural symptomatology […]. The patient voiced two major complaints. The first concerned the feeling of being oneself, and the second his experience of time.” ([140], p. 2).

Although AF’s condition had—to some extent—been successfully treated, not all symptoms had disappeared. Furthermore, and most centrally, the authors explicitly mention that “AF has a rare ability to describe his self and time difficulties” ([140], p. 1). This suggests that most other patients may not even be able to describe remaining symptoms, after the most obvious symptoms have successfully been treated. AF describes his remaining symptoms as follows:

“I do not feel the time,” […] “You see, I can use a metaphor to explain to you… Birds, they have a sense that allows them to orient themselves… a kind of magnetism… It is an innate thing… If they do not have it, they cannot navigate… Me, it’s the time I do not have… I’m like blind to time… but I cannot explain it better… I try to find out how to talk about it… but I can’t manage to explain. It may be the most important thing to understand…” ([140], p. 3).

These statements are highly remarkable: AF has a “rare ability” to describe his problems in some detail, but still struggles to find the right words. At the same time, these problems are extremely important to him. They may be “the most important thing to understand” and yet it is almost impossible for him to explain them.

The case study illustrates at least two things. First, even successful treatments can fail to address crucial aspects of disordered conscious experience. Approaches in CP that mainly seek to dissect spectrum disorders and provide individual treatment predictions are unlikely to improve this situation. Second, there is transformative potential for approaches in CP that seek to account for aspects of disordered conscious experience (as suggested by [98]). If research in CP is beware of ignoring subtle features of subjective experiences, there is thus a chance that it will realise its full potential.

## 7 Conclusion

What revisions of nosology will be suggested by successful clinical applications of computational psychiatry, and by applications of AI in psychiatry? This question is especially relevant because existing research more or less leaves this question open. For instance, Winter et al. [141] propose an “AI ecosystem” to address “fundamental issues regarding sample size, model construction, evaluation practice, and **the conceptualization of mental disorders**” ([141], p. 4, bold emphasis added). However, the way in which mental disorders should be re-conceptualized is not further specified by Winter et al. [141]—apart from the suggestion to use *normative models* [94] to quantify the extent to which individuals deviate from a statistical norm. In particular, it remains undetermined to what extent normative variables (in terms of which deviances are measured) should include not just biological, but also psychological and social variables.

Given methodological constraints, it seems that biological (including neuronal) variables can be measured most easily. Therefore, one might worry that this will reinforce a biological reductionism [40]. However, we argued above that even a focus on biological variables need not lead to a view according to which mental disorders are identical with brain disorders. A more important risk is that biologistic tendencies may ignore important psychosocial factors, such as some features of the conscious experience of patients—although many mental disorders are, to a large extent, disorders of consciousness. Developing normative models that quantify deviances from a statistical norm should therefore also specify what a normal conscious experience is. This could then provide a further “fixed point” for quantitative measures in terms of normal conscious experience—at least in the long run; in the foreseeable future, fixed points may have to be construed in terms of pathological symptoms or endophenotypes of mental illness.

We saw that scientific progress in psychiatry can be regarded as an improvement even when psychosocial factors are ignored. Depending on how “improvement” is defined, however, this can still be ethically problematic. For instance, a reasonable requirement would be that an improvement increase the reliability and validity of diagnostic categories in such a way as to make psychiatric treatments more effective (see [5], p. 20). However, as Barron [138] points out, “there is no consensus on how to measure treatment outcome in psychiatry [142]. For example, would antipsychotic treatment be ‘successful’ if patient R’s hallucinations decrease by 50%? By 90%?” ([138], p. 2). Apart from this, there remains the problem that CP might promote a tendency to exclusively focus on those factors of mental disorders that can be measured, using methods of computational neuroscience, without having to take subjective reports and sociocultural factors into account. Given the status quo in psychiatry, one might argue that *any* improvement—no matter how marginal or restricted—should be regarded as beneficial and desirable, even if central aspects of an ailment remain untreated. However, this should not lead to, for instance, an exclusive focus on developing more effective or personalised therapeutic drugs.

The worries expressed above should be construed as worries about long-term, not short-term effects of CP. In particular, to the extent that translating results of computational neuroscience to clinical practice is successful, one can expect that this will have an impact on how mental disorders are construed. If complemented by research on disturbances of conscious experiences associated with mental disorders, CP may have transformative effects on conceptions of mental disorders that support not overly narrow, but richer and broader views. In particular, it could lead to a deeper understanding of normal and pathological conscious experiences.

In order to reap the benefits of the metaphysical neutrality of computational models, CP should —at least in the long run—be complemented by research on the computational correlates [122] of conscious experiences that go along with mental disorders. This also raises the question what a ‘good,’ ‘normal,’ or ‘pathological’ conscious experience is. Consequently, the ethics of computational psychiatry will also be an ethics of consciousness.

At present, we are far from having a formal account of conscious experience. As mentioned in the introduction, many empirical theories of consciousness make competing claims, and there is still much uncertainty about the neural mechanisms that underwrite ordinary conscious processes (let alone psychopathology). Hence, the suggestion to foster research on the computational correlates of disordered conscious experiences should not be regarded as an invitation to ignore subjective reports. The patient’s perspective will continue to be central for normatively assessing their experienced condition. Computational models offer constructs to better describe and understand elusive aspects of a disordered conscious experience, but the patient will remain the primary authority on whether they are suffering from their condition. Mitigating a disorder may be aided by understanding the neural and computational underpinnings. In this sense, successful applications of CP can be desirable from the point of view of an ethics of consciousness (and the lack of required knowledge about consciousness can be seen as ethically problematic). But such knowledge will not by itself yield a consciousness-ethical account of what a ‘good’, ‘normal’, or ‘pathological’ conscious experience is. Rather, it must build on normative judgments, in order to refine our understanding of disordered conscious experiences.

## CRediT authorship contribution statement

**Wanja Wiese**: Conceptualisation, Writing- Original draft preparation and Editing. **Karl J. Friston**: Writing- Reviewing and Editing.

## Footnotes

1 Gauld et al. [50] even speak of three ‘cultures,’ with digital psychiatry as a further, distinct branch of CP.

2 By this, we do not wish to understate the importance of pharmacological interventions (and other interventions, such as cognitive behavioural therapy). In some cases, such as alcohol use disorder, they may even be more important than ‘folk-psychological wisdom’ would have us think. We thank Matteo Colombo for emphasising this point in personal correspondence.

3 We thank René Baston for pointing us to this study.
